# Supplementary material for: Cell type-specific delivery by modular envelope design
Source: Nat Commun. 2023 Aug 23;14:5141. doi: 10.1038/s41467-023-40788-8 (PMC10447438; doi:10.1038/s41467-023-40788-8)
Supplement: Supplementary file 1 — Supplementary Information [file 41467_2023_40788_MOESM1_ESM.pdf]

Supplementary Information

Cell type-specific delivery by modular envelope design

Daniel Strebinger[1-5], Chris J. Frangieh[1-6], Mirco J. Friedrich[1-5], Guilhem Faure[1-5], Rhiannon K. Macrae[1-5], and Feng Zhang[1-5*]

[1] Howard Hughes Medical Institute
Cambridge, MA 02139, USA

[2] Broad Institute of MIT and Harvard
Cambridge, MA 02142, USA

[3] McGovern Institute for Brain Research
[4] Department of Brain and Cognitive Sciences
[5] Department of Biological Engineering
[6] Department of Electrical Engineering and Computer Science
Massachusetts Institute of Technology
Cambridge, MA 02139, USA

* Corresponding author: zhang@broadinstitute.org (F.Z.)

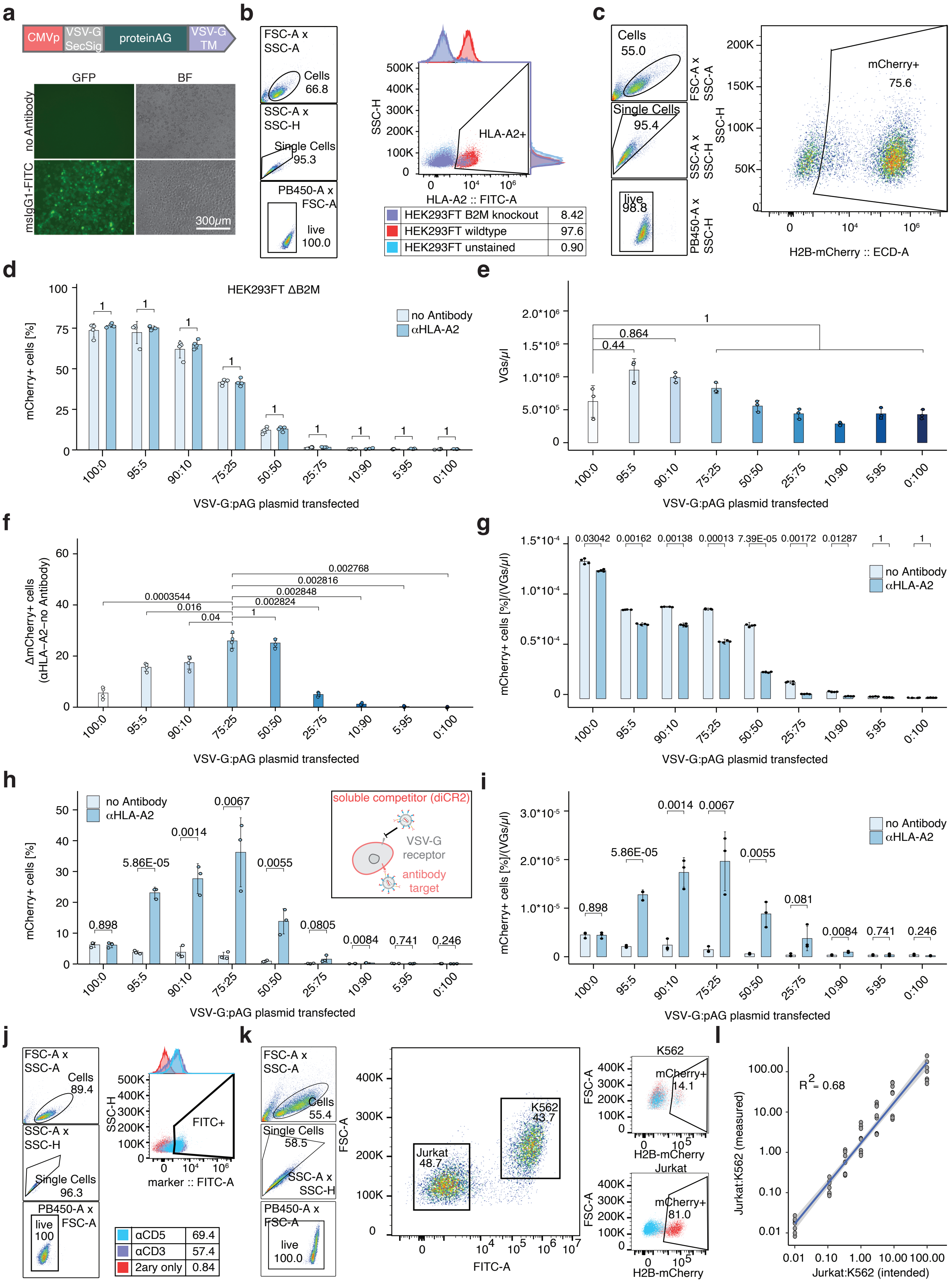
a
CMVp
VSV-G SecSig
proteinAG
VSV-G TM
GFP
BF
no Antibody
msIgG1-FITC
300μm
b
FSC-A x SSC-A
Cells 66.8
SSC-A x SSC-H
Single Cells 95.3
PB450-A x FSC-A
live 100.0
SSC-H
HLA-A2+
HLA-A2 :: FITC-A
HEK293FT B2M knockout 8.42
HEK293FT wildtype 97.6
HEK293FT unstained 0.90
c
Cells 55.0
Single Cells 95.4
live 98.8
mCherry+ 75.6
H2B-mCherry :: ECD-A
d
HEK293FT ΔB2M
mCherry+ cells [%]
no Antibody
αHLA-A2
VSV-G:pAG plasmid transfected
e
VGs/μl
0.864
0.44
f
ΔmCherry+ cells (αHLA-A2–no Antibody)
0.002768
0.002816
0.0003544
0.002848
0.016
0.002824
0.04
g
mCherry+ cells [%]/(VGs/μl)
0.03042
0.00162
0.00138
0.00013
7.39E-05
0.00172
0.01287
h
soluble competitor (diCR2)
VSV-G receptor
antibody target
0.898
5.86E-05
0.0014
0.0067
0.0055
0.0805
0.0084
0.741
0.246
i
0.081
j
Cells 89.4
Single Cells 96.3
live 100
FITC+
marker :: FITC-A
αCD5 69.4
αCD3 57.4
2ary only 0.84
k
Cells 55.4
Single Cells 58.5
live 100.0
Jurkat 48.7
K562 43.7
FITC-A
K562
mCherry+ 14.1
Jurkat
mCherry+ 81.0
H2B-mCherry
l
R^2 = 0.68
Jurkat:K562 (measured)
Jurkat:K562 (intended)

Supplementary Figure 1: **Development of DIRECTED**

a, (Top) Schematic showing details of protein AG (pAG) construct. (CMVp - CMV promoter, VSV-G SecSig - secretion signal of VSV-G [MKCLLYLAFLFIGVNC], VSV-G TM - Transmembrane domain of VSV-G [NPIELVEGWFSSWKSSIASFFFIIGLIIGLFLVLRVGIHLCIKLKHTKKRQIYTDIEMNRLGK]) (Bottom) Labeling of pAG transfected HEK293FT cells with a FITC-labelled antibody (representative of 3 experiments, Scale bar: 300µm). **b,** Gating strategy and surface expression of HLA-A2 in wild-type and ΔB2M HEK293FT cells. **c,** Gating strategy for HEK293FT cells after transduction to detect H2B-mCherry transgene (related to Figure 1b,c,d, and Figure 2b,c). **d,** Performance of VSV-G particles co-expressing pAG at different ratios without antibody or in the presence of αHLA-A2 antibody on ΔB2M HEK293FT. (N=4 for each condition) **e,** Physical titer of lentiviral particles produced upon transfection of different ratios of VSV-G:pAG plasmid during lentiviral production. (N=3 per condition) **f,** Difference in %mCherry+ cells in presence of an αHLA-A2 antibody minus the percentage without antibody. (related to Figure 1b, N=4 per condition) **g,** Transduction efficiency (%mCherry+ cells) normalized to the physical titer for VLPs produced at different ratios of VSV-G:pAG with or without αHLA-A2 antibody (N=4 per condition). **h,** Transduction efficiency of HEK293FT cells as %mCherry+ cells upon transduction with wildtype VSV-G and pAG pseudotyped vectors at the indicated ratios in the presence of a competitor for VSV-G:LDL-R receptor interaction (diCR2) and in the absence or presence of an αHLA-A2 targeting antibody (N=3 per condition). **i,** Transduction efficiency (%mCherry+ cells) normalized to physical titer for lentiviral particles produced at different VSV-G:pAG ratios with or without αHLA-A2 antibody in the presence of soluble diCR2 (N=3 per condition). **j,** Gating strategy and surface level expression of CD3 and CD5 on Jurkat E6 cells. **k,** Gating strategy for the Jurkat and K562 co-culture experiments. **l,** Plot showing intended versus measured Jurkat:K562 ratio for the co-culture experiments. (related to Figure 1f, N=8 per condition). Analyses in panels d, e, f, and g used two-sided Welch's t test with Bonferroni correction. Analyses in panels h, and i, use an ANOVA followed by Dunnett's post-hoc test with Bonferroni correction. Data are presented as mean ± standard deviation. Source data are provided as a Source Data file.

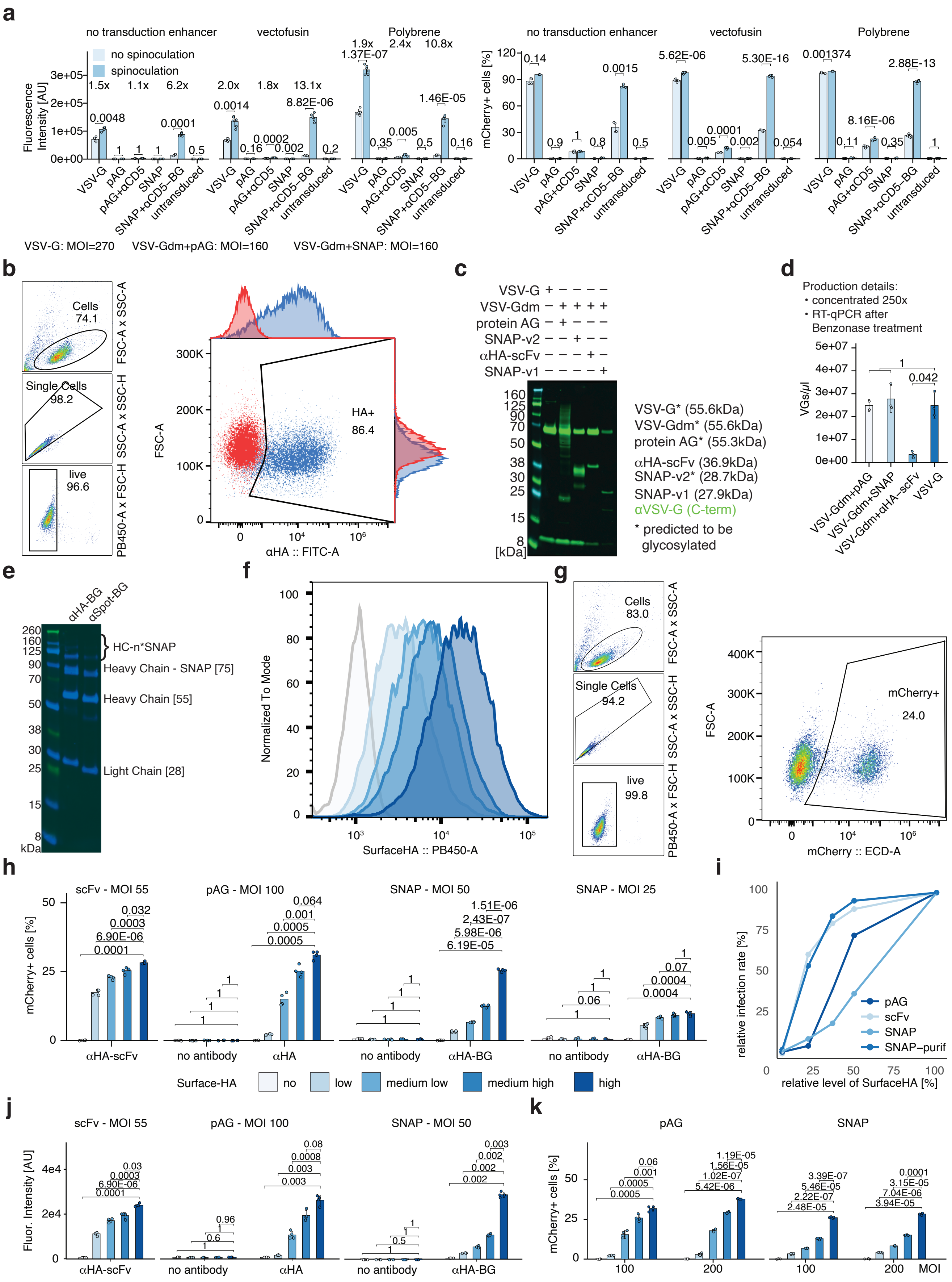

a
no transduction enhancer
vectofusin
Polybrene
no spinoculation
spinoculation
Fluorescence Intensity [AU]
mCherry+ cells [%]
VSV-G
pAG
pAG+αCD5
SNAP
SNAP+αCD5-BG
untransduced
VSV-G: MOI=270
VSV-Gdm+pAG: MOI=160
VSV-Gdm+SNAP: MOI=160
b
Cells 74.1
FSC-A x SSC-A
Single Cells 98.2
SSC-A x SSC-H
live 96.6
PB450-A x FSC-H
FSC-A
HA+ 86.4
αHA :: FITC-A
c
VSV-G
VSV-Gdm
protein AG
SNAP-v2
αHA-scFv
SNAP-v1
[kDa]
VSV-G* (55.6kDa)
VSV-Gdm* (55.6kDa)
protein AG* (55.3kDa)
αHA-scFv (36.9kDa)
SNAP-v2* (28.7kDa)
SNAP-v1 (27.9kDa)
αVSV-G (C-term)
* predicted to be glycosylated
d
Production details:
• concentrated 250x
• RT-qPCR after Benzonase treatment
VGs/µl
VSV-Gdm+pAG
VSV-Gdm+SNAP
VSV-Gdm+αHA-scFv
VSV-G
e
αHA-BG
αSpot-BG
HC-n*SNAP
Heavy Chain - SNAP [75]
Heavy Chain [55]
Light Chain [28]
kDa
f
Normalized To Mode
SurfaceHA :: PB450-A
g
Cells 83.0
FSC-A x SSC-A
Single Cells 94.2
SSC-A x SSC-H
live 99.8
PB450-A x FSC-H
FSC-A
mCherry+ 24.0
mCherry :: ECD-A
h
scFv - MOI 55
pAG - MOI 100
SNAP - MOI 50
SNAP - MOI 25
mCherry+ cells [%]
αHA-scFv
no antibody
αHA
αHA-BG
Surface-HA
no
low
medium low
medium high
high
i
relative infection rate [%]
relative level of SurfaceHA [%]
pAG
scFv
SNAP
SNAP–purif
j
scFv - MOI 55
pAG - MOI 100
SNAP - MOI 50
Fluor. Intensity [AU]
αHA-scFv
no antibody
αHA
αHA-BG
k
pAG
SNAP
mCherry+ cells [%]
MOI

Supplementary Figure 2: **Targeting strategies for DIRECTED and receptor level impact**

a, (Left) Impact of spinoculation and transduction enhancers on the transduction efficiency (measured as the fluorescence intensity of mCherry in all live cells) of Jurkat E6 cells for wildtype VSV-G and the pAG and SNAP programmable DIRECTED envelopes. (Right) Transduction efficiency of the same cells as presented in the left panel expressed as percentage of mCherry+ cells. (N=4 for cells in the absence of transduction enhancers; N=6 for vectofusin and polybrene experiments) **b,** Gating strategy for surface staining of Jurkat+surface-HA cells with an αHA antibody. **c,** Western blot analysis of lentiviral particles produced with the indicated components probed with an αVSV-G antibody. (Representative example of 3 blots) **d,** Physical titer of conventional (VSV-G) and DIRECTED lentiviral particles (using the SNAP, pAG, or scFv strategy; N=3 per condition). **e,** Western blot analysis of BG labelled antibodies after co-incubation with purified SNAP protein. (Representative Example of 3 blots) **f,** Histogram showing the distribution of surface-HA expression levels in the four bins (low, medium low, medium high, and high) after sorting cells for surface-HA levels as determined by flow cytometry upon staining with αHA-PB450. **g,** Gating strategy to analyze transduction efficiency of Jurkat+surface-HA cells (related to Figure 1e, and Figure 2a). **h,** Percentage showing the transduction efficiency of the different surface-HA populations for the scFv, pAG, and SNAP DIRECTED strategies. (related to Figure 2a; N=4 for each condition) **i,** Plot showing the relative level of surface-HA (x-axis) versus the relative infection rate for each of the strategies presented in Figure 2a. **j,** Fluorescence intensity in all live cells upon transduction for the four bins with different surface-HA expression and wildtype cells. (N=4 per condition). **k,** Comparison of the transduction efficiency of pAG (left) or SNAP (right) DIRECTED lentiviral vectors targeting surface-HA on cells sorted for different receptor level expression at different MOI. (excess antibody was not removed for the SNAP strategy, N=4 for each condition). For analyses in panels a, d, h, j, and k a two-sided Welch's t test with Bonferroni correction was performed. Data are presented as mean ± standard deviation. Source data are provided as a Source Data file.

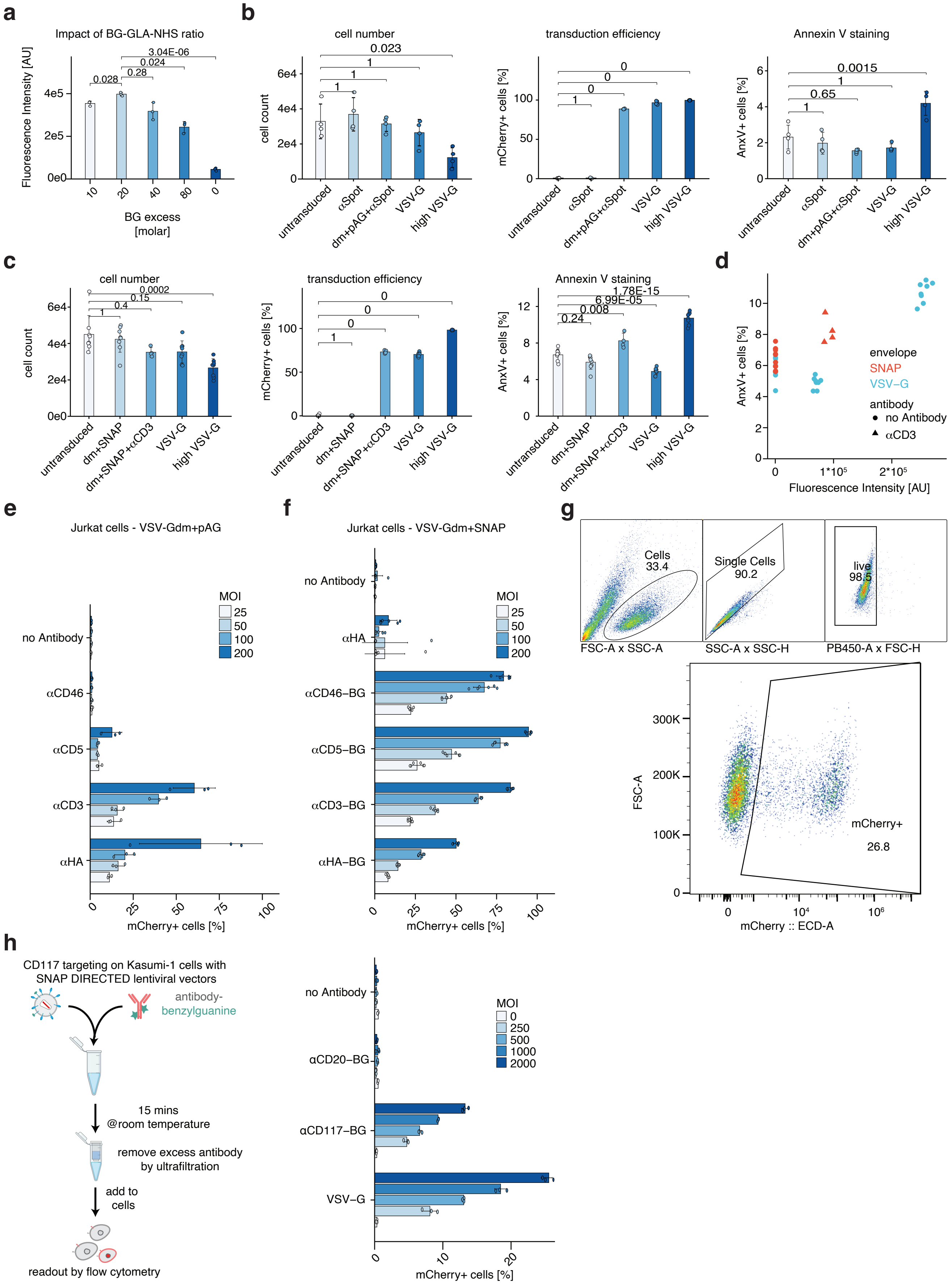
a
Impact of BG-GLA-NHS ratio
Fluorescence Intensity [AU]
BG excess [molar]
3.04E-06
0.024
0.28
0.028
b
cell number
cell count
0.023
transduction efficiency
mCherry+ cells [%]
Annexin V staining
AnxV+ cells [%]
0.0015
0.65
untransduced
αSpot
dm+pAG+αSpot
VSV-G
high VSV-G
c
cell number
0.0002
0.15
0.4
transduction efficiency
Annexin V staining
1.78E-15
6.99E-05
0.008
0.24
untransduced
dm+SNAP
dm+SNAP+αCD3
VSV-G
high VSV-G
d
AnxV+ cells [%]
envelope
SNAP
VSV–G
antibody
no Antibody
αCD3
Fluorescence Intensity [AU]
e
Jurkat cells - VSV-Gdm+pAG
MOI
no Antibody
αCD46
αCD5
αCD3
αHA
mCherry+ cells [%]
f
Jurkat cells - VSV-Gdm+SNAP
no Antibody
αHA
αCD46–BG
αCD5–BG
αCD3–BG
αHA–BG
mCherry+ cells [%]
g
Cells
33.4
Single Cells
90.2
live
98.5
FSC-A x SSC-A
SSC-A x SSC-H
PB450-A x FSC-H
FSC-A
mCherry+
26.8
mCherry :: ECD-A
h
CD117 targeting on Kasumi-1 cells with SNAP DIRECTED lentiviral vectors
antibody-benzylguanine
15 mins @room temperature
remove excess antibody by ultrafiltration
add to cells
readout by flow cytometry
no Antibody
αCD20–BG
αCD117–BG
VSV–G
MOI
mCherry+ cells [%]

Supplementary Figure 3: **Specificity of DIRECTED**

a, Impact of different ratios of molar excess of benzygluanine NHS ester (BG-GLA-NHS) to αSpot antibody on the transduction efficiency of HEK293FT cells engineered to express a surface-Spot receptor. Transduction efficiency is evaluated as the fluorescence intensity of all live cells. (N=3 per condition) **b,** Cell number (left), Transduction efficiency (middle), and fraction of Annexin V positive cells (right) upon transduction of HEK293FT + surface-Spot cells with αSpot pAG-DIRECTED (MOI=300), VSV-G (MOI=300), or high VSV-G (MOI=10.000) lentiviral vectors. (N=4 per condition) **c,** Cell number (left), Transduction efficiency (middle), and fraction of Annexin V positive cells (right) upon transduction of Jurkat E6 cells with SNAP-DIRECTED (no antibody, MOI=50), αCD3-BG SNAP-DIRECTED (MOI=50), VSV-G (MOI=50), or high VSV-G (MOI=400) lentiviral vectors. (N=4 for dm+SNAP+αCD3, N=8 for all other conditions) **d,** Scatter plot showing the Fluorescence Intensity in all live cells (to indicate transduction efficiency) versus the fraction of Annexin V positive cells for the conditions presented in panel c. **e,** Performance of pAG-DIRECTED lentiviral vectors on Jurkat+surface-HA cells targeting HA, CD5, CD46, or CD3 determined as the percentage of mCherry+ cells at different MOI. (N=3 per condition) **f,** Performance of SNAP-DIRECTED lentiviral vectors on Jurkat+surface-HA cells using αHA-BG, αCD5-BG, αCD46-BG, αCD3-BG, or αHA determined as the percentage of mCherry+ cells at different MOI. (excess antibody was not removed, N=6 per condition) **g,** Gating strategy to analyze transduction efficiency of Kasumi-1 cells. **h,** (left) Schematic for the removal of excess antibody using ultrafiltration. (right) Transduction efficiency of SNAP-DIRECTED particles on Kasumi-1 cells using no antibody, αCD117-BG, or αCD20-BG, or wild-type VSV-G lentiviral particles at different MOI. (N=3 per condition) A part of the schematics in panel 3h were prepared using icons from biorender (biorender.com). Data are presented as mean ± standard deviation. Source data are provided as a Source Data file. For analyses in panel a a two-sided Welch's t test with Bonferroni correction was performed. Analyses in panels b,c a Kruskal test was used, followed by a Dunnett's post-hoc test if significant.

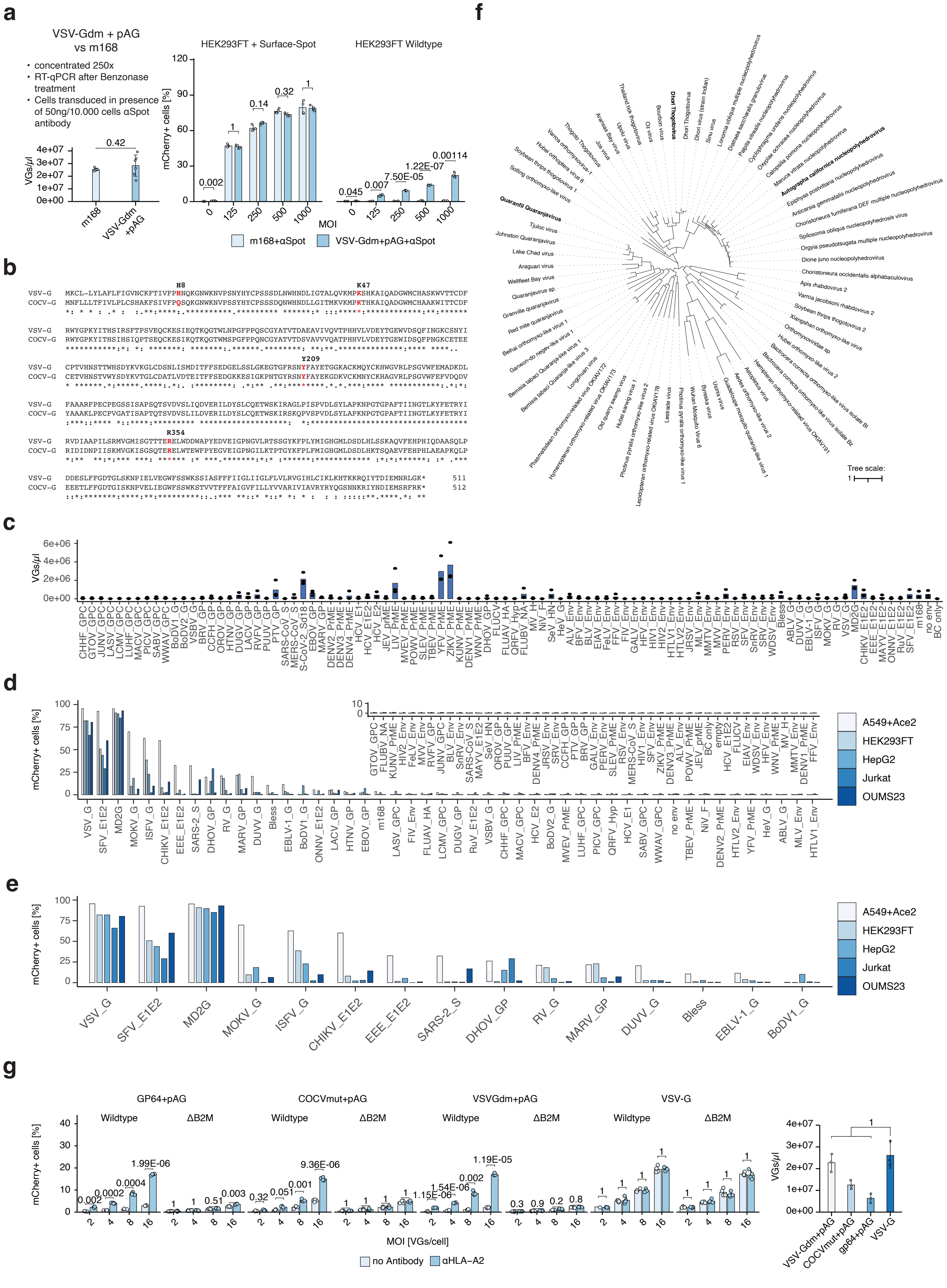

a
VSV-Gdm + pAG vs m168
concentrated 250x
RT-qPCR after Benzonase treatment
Cells transduced in presence of 50ng/10.000 cells αSpot antibody
VGs/µl
m168
VSV-Gdm +pAG
0.42
HEK293FT + Surface-Spot
HEK293FT Wildtype
mCherry+ cells [%]
MOI
m168+αSpot
VSV-Gdm+pAG+αSpot
b
VSV-G
COCV-G
H8
K47
Y209
R354
f
Tree scale: 1
c
VGs/µl
d
mCherry+ cells [%]
A549+Ace2
HEK293FT
HepG2
Jurkat
OUMS23
e
mCherry+ cells [%]
VSV_G
SFV_E1E2
MD2G
MOKV_G
ISFV_G
CHIKV_E1E2
EEE_E1E2
SARS-2_S
DHOV_GP
RV_G
MARV_GP
DUVV_G
Bless
EBLV-1_G
BoDV1_G
g
GP64+pAG
COCVmut+pAG
VSVGdm+pAG
VSV-G
Wildtype
ΔB2M
mCherry+ cells [%]
MOI [VGs/cell]
no Antibody
αHLA−A2
VGs/µl
VSV-Gdm+pAG
COCVmut+pAG
gp64+pAG
VSV-G

Supplementary Figure 4: **Exploration of the natural diversity of fusogens and their compatibility with DIRECTED**

a, Comparison of m168 and VSV-Gdm+pAG DIRECTED particles targeting a synthetic surface-Spot receptor on HEK293FT cells. (left) Titers of particles as determined by RT-qPCR for the lentiviral genome expressed at viral genomes/µl. (N=6 per condition) (right) Infection rate expressed as percentage of mCherry+ cells on HEK293FT + surface-Spot or wildtype HEK293FT cells in the presence of an αSpot antibody at different MOI (N=4 for each condition). **b,** Sequence alignment of VSV-G and Cocal virus G (COCV-G) with the key residues responsible for VSV-G/LDL-R interaction highlighted in red. The mutant version of Cocal Virus G has all 4 highlighted residues mutated to Alanine. **c,** Physical titer of pseudotyped particles produced with different wildtype fusogens expressed as viral genomes per microliter from plain supernatants (N=3 per condition). **d,** Evaluation of pseudoviruses with different viral fusogens on a panel of 5 human cell lines (A549+Ace2, HEK293FT, HepG2, Jurkat E6, and OUMS23). **e,** Detailed view of the 15 most efficient fusogens. **f,** Phylogenetic tree of orthomyxoviral envelope proteins using Quaranjafil quaranjavirus (hypothetical protein) as a seed. **g,** Comparison of the transduction efficiency as percentage of mCherry+ cells for GP64+pAG, COCVmut+pAG, VSV-Gdm+pAG, and wildtype VSV-G at the same MOIs in the presence or absence of an αHLA-A2 antibody on wildtype HEK293FT cells or ΔB2M HEK293FT cells. (N=4 per condition). Data are presented as mean ± standard deviation. Source data are provided as a Source Data file. For analyses in panels a, and g a two-sided Welch's t test with Bonferroni correction was performed.

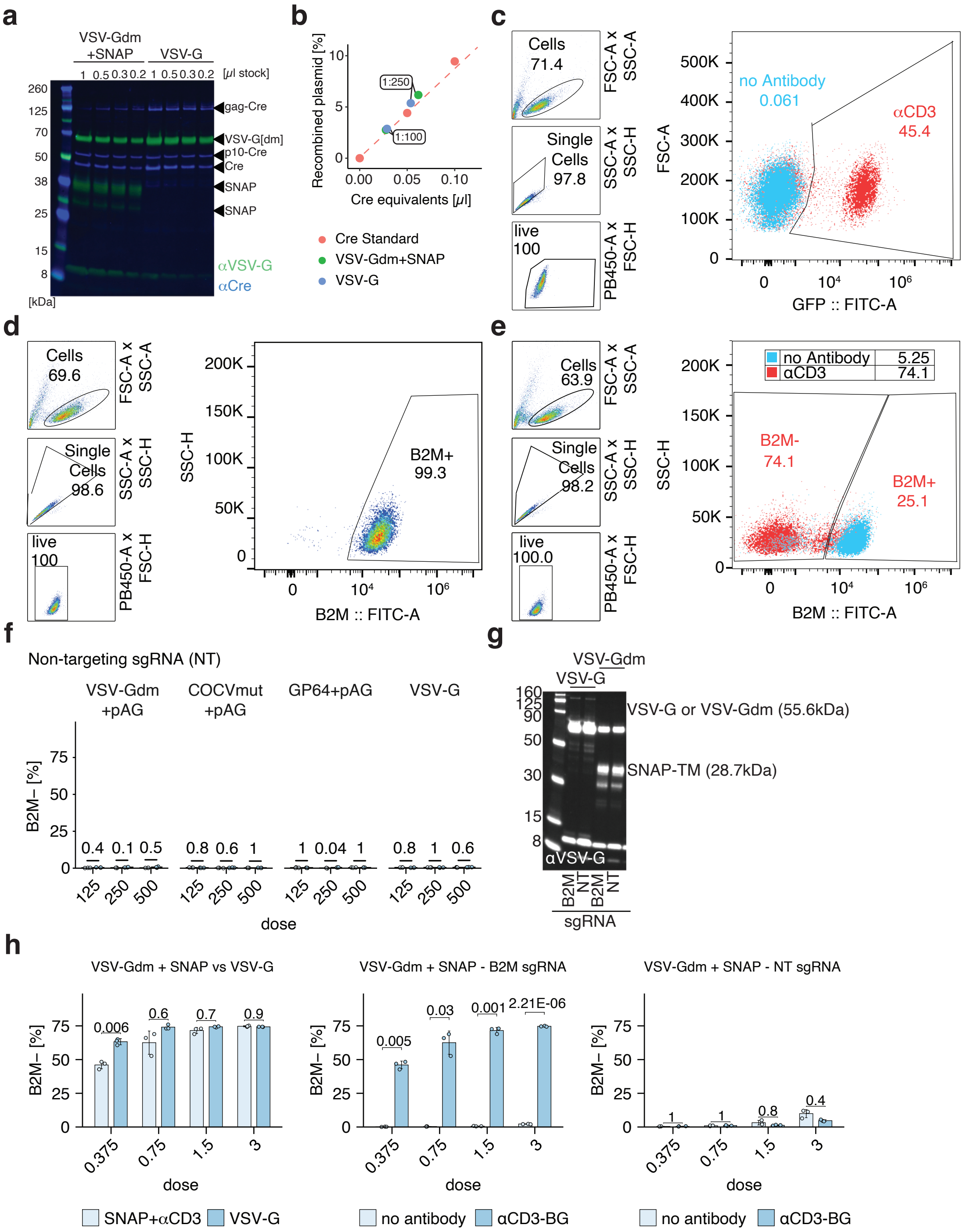

a
VSV-Gdm +SNAP
VSV-G
1 0.5 0.3 0.2 1 0.5 0.3 0.2 [μl stock]
260
125
70
50
38
25
15
8
[kDa]
gag-Cre
VSV-G[dm]
p10-Cre
Cre
SNAP
SNAP
αVSV-G
αCre
b
Recombined plasmid [%]
Cre equivalents [μl]
1:250
1:100
Cre Standard
VSV-Gdm+SNAP
VSV-G
c
Cells 71.4
FSC-A x SSC-A
Single Cells 97.8
SSC-A x SSC-H
live 100
PB450-A x FSC-H
FSC-A
no Antibody 0.061
αCD3 45.4
GFP :: FITC-A
d
Cells 69.6
Single Cells 98.6
live 100
SSC-H
B2M+ 99.3
B2M :: FITC-A
e
Cells 63.9
Single Cells 98.2
live 100.0
no Antibody 5.25
αCD3 74.1
B2M- 74.1
B2M+ 25.1
B2M :: FITC-A
f
Non-targeting sgRNA (NT)
VSV-Gdm +pAG
COCVmut +pAG
GP64+pAG
VSV-G
B2M- [%]
0.4 0.1 0.5 0.8 0.6 1 1 0.04 1 0.8 1 0.6
dose
g
VSV-Gdm
VSV-G
VSV-G or VSV-Gdm (55.6kDa)
SNAP-TM (28.7kDa)
αVSV-G
B2M NT B2M NT
sgRNA
h
VSV-Gdm + SNAP vs VSV-G
0.006 0.6 0.7 0.9
VSV-Gdm + SNAP - B2M sgRNA
0.005 0.03 0.001 2.21E-06
VSV-Gdm + SNAP - NT sgRNA
1 1 0.8 0.4
B2M- [%]
dose
SNAP+αCD3
VSV-G
no antibody
αCD3-BG

Supplementary Figure 5: **DIRECTED is compatible with modalities that allow protein or RNP delivery** **a,** Western blot analysis of CreVLPs produced with VSV-Gdm+SNAP or VSV-G and probed with αVSV-G or αCre antibodies. (Representative example of 3 blots) **b,** Cre content versus recombination efficiency of in vitro Cre reactions. Indicated labels represent the dilution factors used for the corresponding CreVLPs. **c,** Gating strategy for the analysis of GFP expression in Jurkat E6 Cre reporter cells upon treatment with CreVLPs. **d,** Gating strategy and analysis of B2M expression on Jurkat E6 cells. **e,** Gating strategy to analyze the B2M expression on Jurkat E6 cells after treatment with DIRECTED-CreVLPs. **f,** Analysis of the loss of B2M expression on Jurkat E6 cells treated with eVLPs with the indicated envelopes packaging non-targeting sgRNAs (NT). (N=3 for each condition) **g,** Western blot analysis of VSV-G or VSV-Gdm+SNAP Cas9-RNP containing particles probed with αVSV-G antibody. (Representative example of 3 blots) **h,** (left) Comparison of wildtype VSV-G or αCD3 VSV-Gdm+SNAP particles delivering B2M targeting Cas9-RNP. (middle) Performance of SNAP DIRECTED VLPs in the absence or presence of a CD3-targeting antibody on the loss of B2M surface expression level. (right) Analysis of the loss of B2M expression on Jurkat E6 cells treated with eVLPs with VSV-Gdm+SNAP packaging a non-targeting sgRNA (NT) in the absence or presence of a CD3-targeting antibody. (excess antibody was not removed, N=3 per condition). Data are presented as mean ± standard deviation. Source data are provided as a Source Data file. For analyses in panels f, and h a two-sided Welch's t test with Bonferroni correction was performed.

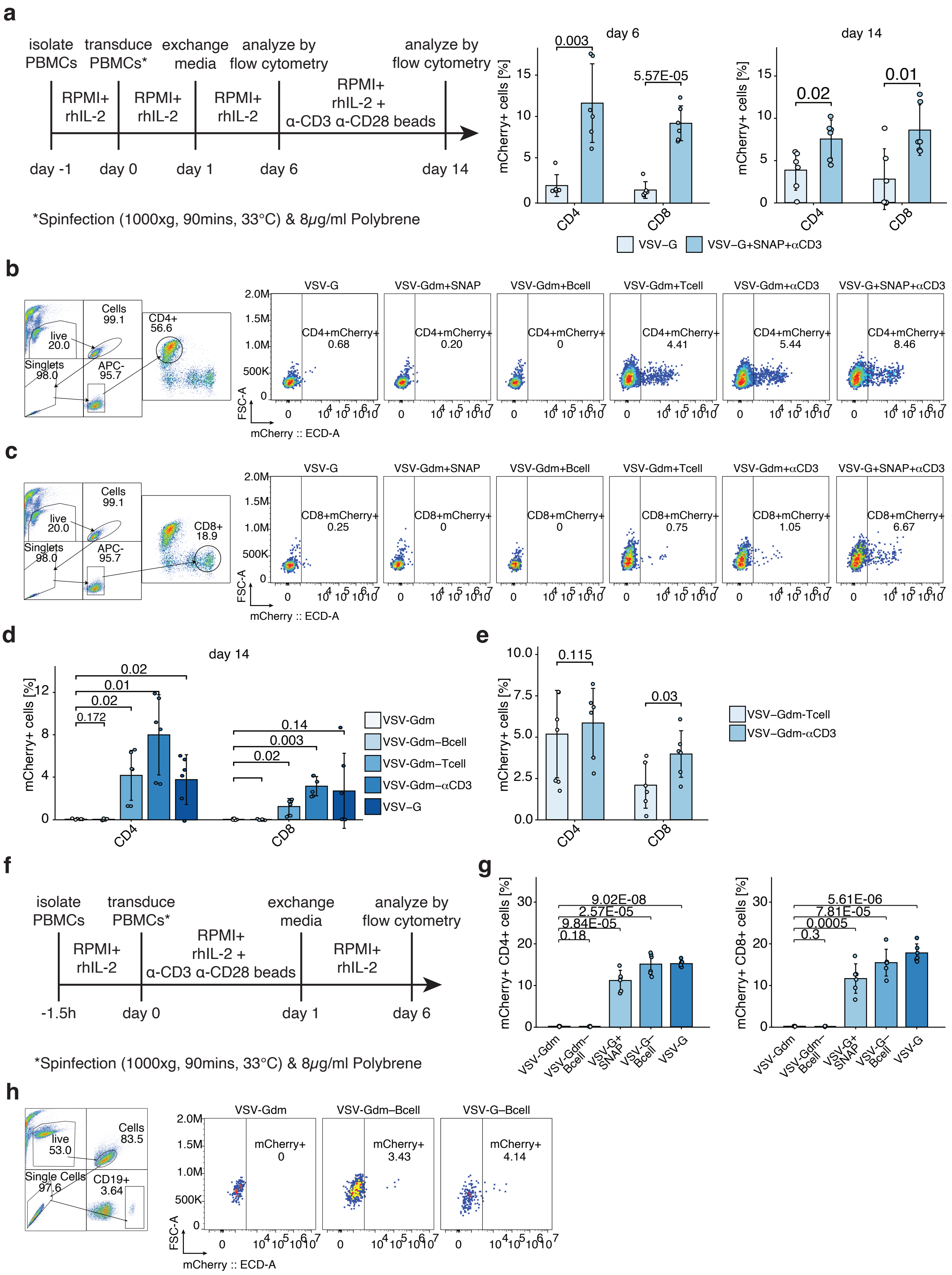

a
isolate PBMCs
transduce PBMCs*
exchange media
analyze by flow cytometry
analyze by flow cytometry
RPMI+ rhIL-2
RPMI+ rhIL-2
RPMI+ rhIL-2
RPMI+ rhIL-2 + α-CD3 α-CD28 beads
day -1
day 0
day 1
day 6
day 14
*Spinfection (1000xg, 90mins, 33°C) & 8μg/ml Polybrene
day 6
day 14
mCherry+ cells [%]
0.003
5.57E-05
0.02
0.01
CD4
CD8
VSV−G
VSV−G+SNAP+αCD3
b
Cells 99.1
live 20.0
Singlets 98.0
APC- 95.7
CD4+ 56.6
VSV-G
VSV-Gdm+SNAP
VSV-Gdm+Bcell
VSV-Gdm+Tcell
VSV-Gdm+αCD3
VSV-G+SNAP+αCD3
CD4+mCherry+ 0.68
CD4+mCherry+ 0.20
CD4+mCherry+ 0
CD4+mCherry+ 4.41
CD4+mCherry+ 5.44
CD4+mCherry+ 8.46
FSC-A
mCherry :: ECD-A
c
Cells 99.1
live 20.0
Singlets 98.0
APC- 95.7
CD8+ 18.9
CD8+mCherry+ 0.25
CD8+mCherry+ 0
CD8+mCherry+ 0
CD8+mCherry+ 0.75
CD8+mCherry+ 1.05
CD8+mCherry+ 6.67
FSC-A
mCherry :: ECD-A
d
day 14
mCherry+ cells [%]
0.02
0.01
0.02
0.172
0.14
0.003
0.02
CD4
CD8
VSV-Gdm
VSV-Gdm−Bcell
VSV-Gdm−Tcell
VSV-Gdm−αCD3
VSV-G
e
mCherry+ cells [%]
0.115
0.03
CD4
CD8
VSV−Gdm-Tcell
VSV−Gdm-αCD3
f
isolate PBMCs
transduce PBMCs*
exchange media
analyze by flow cytometry
RPMI+ rhIL-2
RPMI+ rhIL-2 + α-CD3 α-CD28 beads
RPMI+ rhIL-2
-1.5h
day 0
day 1
day 6
*Spinfection (1000xg, 90mins, 33°C) & 8μg/ml Polybrene
g
mCherry+ CD4+ cells [%]
9.02E-08
2.57E-05
9.84E-05
0.18
mCherry+ CD8+ cells [%]
5.61E-06
7.81E-05
0.0005
0.3
VSV-Gdm
VSV-Gdm−Bcell
VSV-G+SNAP
VSV-G−Bcell
VSV-G
h
Cells 83.5
live 53.0
Single Cells 97.6
CD19+ 3.64
VSV-Gdm
VSV-Gdm−Bcell
VSV-G−Bcell
mCherry+ 0
mCherry+ 3.43
mCherry+ 4.14
FSC-A
mCherry :: ECD-A

Supplementary Figure 6: **Targeting of specific cell types in PBMCs with DIRECTED**

a, (left) Experimental setup for targeting of T cells in PBMCs. (right) Delivery efficiency of H2B-mCherry transgene by VSV-G and VSV-G+SNAP+αCD3 particles to primary human T cells in PBMCs from 3 donors measured at day 6 and day 14 post infection. (N=2 infections per donor/3 donors) **b,** (left) Gating strategy to analyze H2B-mCherry expression in CD4+ T cells. (right) Sample data from T cell targeting experiment in CD4+ T cells. **c,** (left) Gating strategy to analyze the H2B-mCherry expression in CD8+ T cells. (right) Sample data from the T cell targeting experiment in CD8+ T cells. **d,** Delivery efficiency of H2B-mCherry transgene to primary human T cells in PBMCs from 3 donors by VSV-G and VSV-Gdm+SNAP lentiviral vectors in absence of antibody (VSV-Gdm) or functionalized with B cell targeting ligands (αCD19-BG, MegaCD40L-BG; VSV-Gdm-Bcell), T cell targeting ligands (αCD3-BG, αCD28-BG, αCD4-BG; VSV-Gdm-Tcell), or αCD3 (VSV-Gdm-αCD3-BG) measured on day 14 post infection. Shown is %mCherry+ cells of CD4+ T cells (left) or CD8+ T cells (right). VSV-G and VSV-Gdm: MOI 500, other variants: MOI 250. (N=2 infections per donor/3 donors) **e,** Comparison of transduction efficiency of VSV-Gdm-Tcell and VSV-Gdm-αCD3 on CD4+ and CD8+ T cells on day 6. (N=2 infections per donor/3 donors) **f,** Experimental setup for B cell targeting in PBMCs. **g,** Off-target delivery of H2B-mCherry transgene to primary human T cells in PBMCs from 3 donors by VSV-G, VSV-G+SNAP and VSV-Gdm+SNAP lentivectors. VSV-Gdm and VSV-G+SNAP denote particles in the absence of targeting ligands. VSV-Gdm-Bcell and VSV-G-Bcell denote particles functionalized with B cell targeting ligands (αCD19-BG, MegaCD40L-BG). VSV-G, VSV-G+SNAP and VSV-Gdm: MOI 3500, VSV-Gdm-Bcell, and VSV-G-Bcell: MOI 1750. (N=2 infections per donor/3 donors) **h,** (left) gating strategy to analyze H2B-mCherry expression in CD19+ B cells. (right) sample data from the B cell targeting experiment in CD19+ B cells. Analyses in panels a, and g, used two-sided Welch's t test and analysis in panel e, used two-sided Welch's t test. Analyses in panel d, used a paired, two-sided Welch's t test with BH correction. Data are presented as mean ± standard deviation. Source data are provided as a Source Data file.

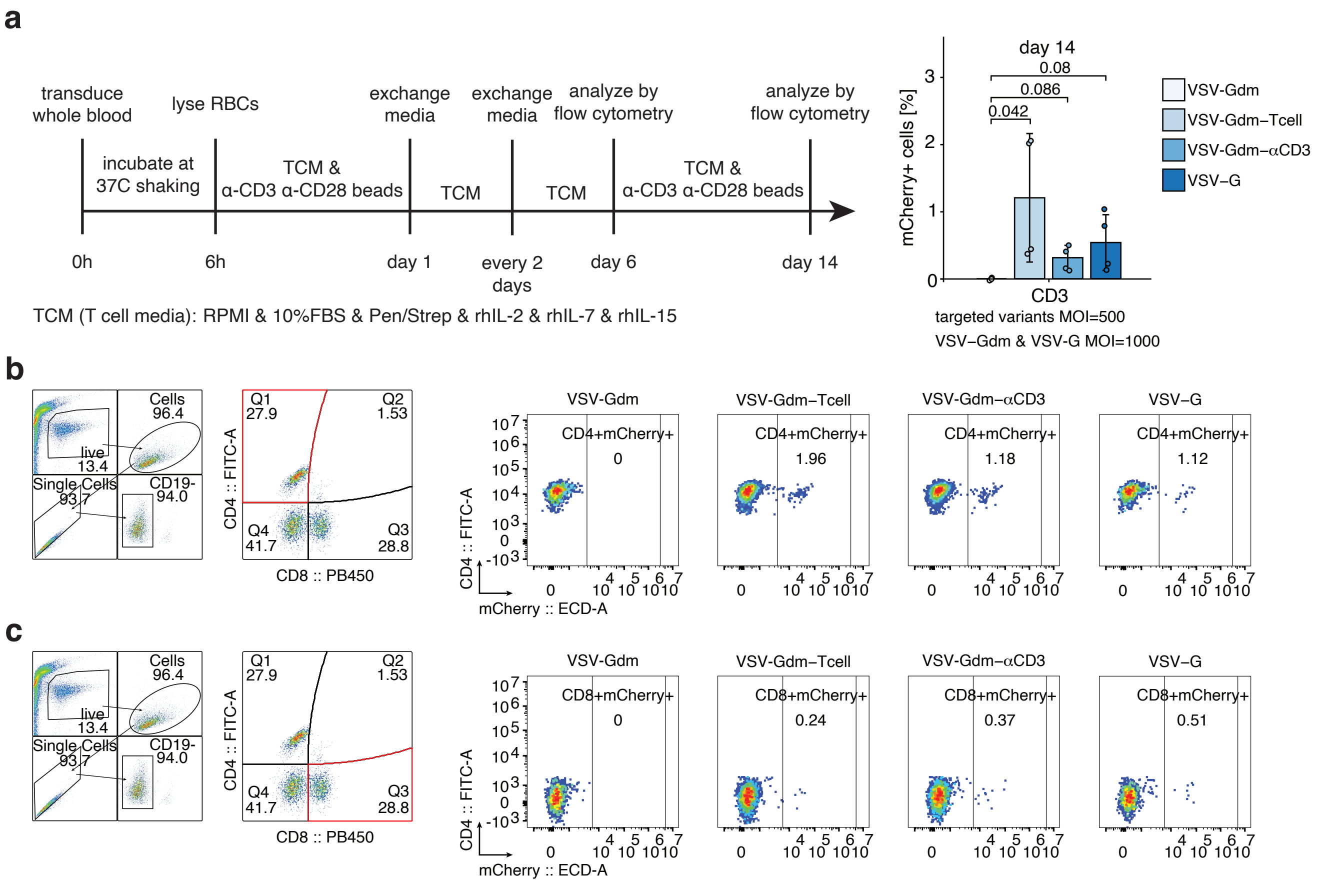
a
transduce whole blood
lyse RBCs
exchange media
exchange media
analyze by flow cytometry
analyze by flow cytometry
incubate at 37C shaking
TCM & α-CD3 α-CD28 beads
TCM
TCM
TCM & α-CD3 α-CD28 beads
0h
6h
day 1
every 2 days
day 6
day 14
TCM (T cell media): RPMI & 10%FBS & Pen/Strep & rhIL-2 & rhIL-7 & rhIL-15
day 14
mCherry+ cells [%]
0.08
0.086
0.042
CD3
targeted variants MOI=500
VSV−Gdm & VSV-G MOI=1000
VSV-Gdm
VSV-Gdm−Tcell
VSV-Gdm−αCD3
VSV−G
b
Cells 96.4
live 13.4
Single Cells 93.7
CD19- 94.0
Q1 27.9
Q2 1.53
Q4 41.7
Q3 28.8
CD4 :: FITC-A
CD8 :: PB450
VSV-Gdm
VSV-Gdm−Tcell
VSV-Gdm−αCD3
VSV−G
CD4+mCherry+ 0
CD4+mCherry+ 1.96
CD4+mCherry+ 1.18
CD4+mCherry+ 1.12
mCherry :: ECD-A
c
Cells 96.4
live 13.4
Single Cells 93.7
CD19- 94.0
Q1 27.9
Q2 1.53
Q4 41.7
Q3 28.8
CD4 :: FITC-A
CD8 :: PB450
VSV-Gdm
VSV-Gdm−Tcell
VSV-Gdm−αCD3
VSV−G
CD8+mCherry+ 0
CD8+mCherry+ 0.24
CD8+mCherry+ 0.37
CD8+mCherry+ 0.51
mCherry :: ECD-A

Supplementary Figure 7: **DIRECTED allows targeting of T cells in whole blood**

a, (left) Flowchart showing the experimental setup for T cell targeting in whole blood. (right) Delivery efficiency of H2B-mCherry transgene to primary human T cells in whole blood from 2 individual donors by wild-type VSV-G and VSV-Gdm+SNAP pseudotyped lentiviral vectors in the absence of antibody (VSV-Gdm) or functionalized with T cell targeting ligands (αCD3-BG, αCD28-BG, αCD4-BG; VSV-Gdm-Tcell), or αCD3 (VSV-Gdm-αCD3-BG) measured on day 14 post infection. Shown is the percentage of mCherry+ cells of CD3+ T cells. Wild-type VSV-G and VSV-Gdm were used at an MOI of 1000, whereas VSV-Gdm-Bcell, VSV-Gdm-Tcell, and VSV-Gdm-αCD3 were used at an MOI of 500. MOIs were calculated estimating 5000 leukocytes per μl of whole blood. (N=2 independent infections per donor for 2 donors) **b,** (left) Gating strategy for the analysis of H2B-mCherry expression in CD4+ T cells. (right) Sample data from the T cell targeting experiment in whole blood for the indicated conditions in CD4+ T cells. **c,** (left) Gating strategy for the analysis of H2B-mCherry expression in CD8+ T cells. (right) Sample data from the T cell targeting experiment in whole blood for the indicated conditions in CD8+ T cells. For analyses in panel a a paired, two-sided Welch's t test was used. Data are presented as mean ± standard deviation. Source data are provided as a Source Data file.

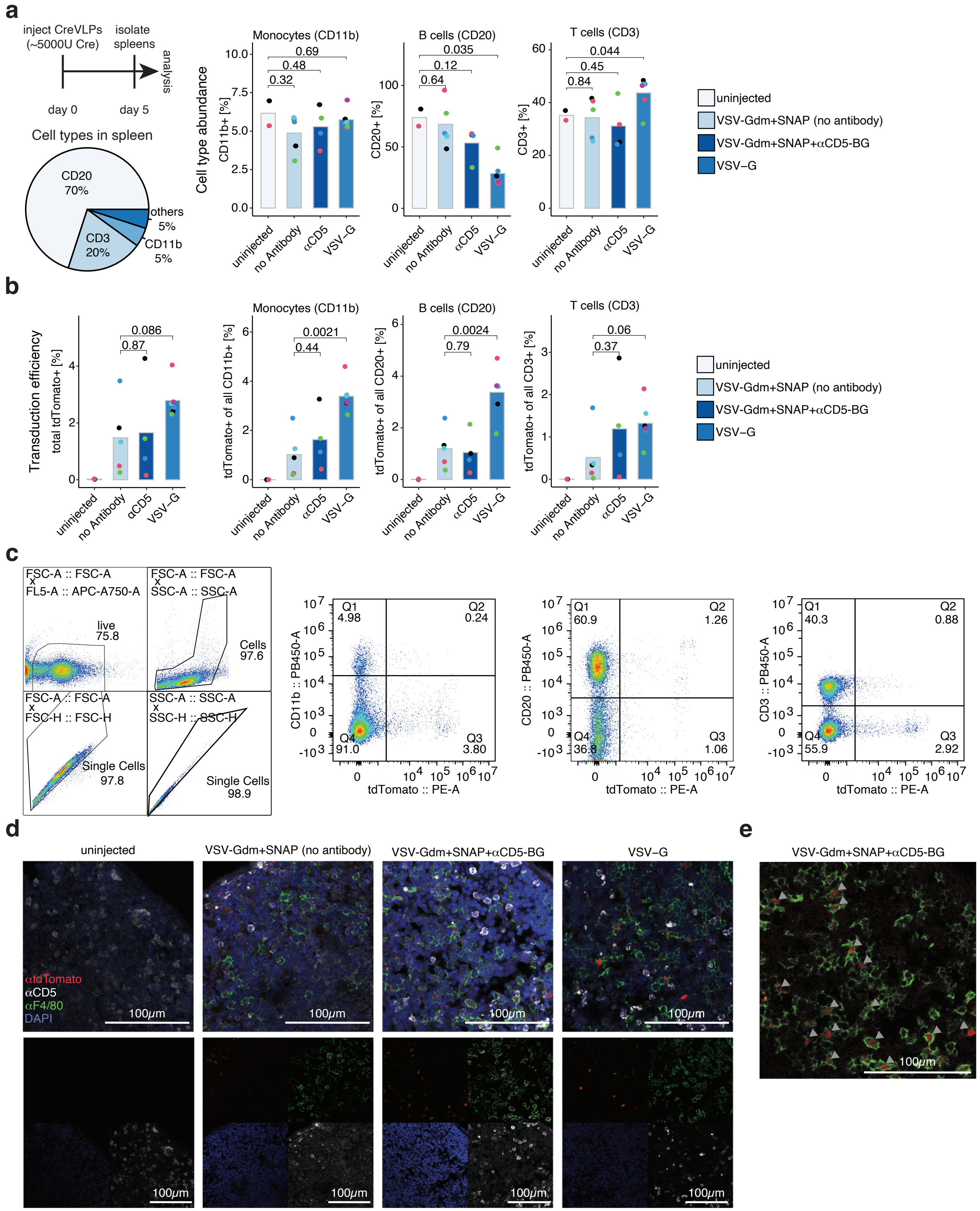

a
inject CreVLPs (~5000U Cre)
isolate spleens
analysis
day 0
day 5
Cell types in spleen
CD20 70%
CD3 20%
others 5%
CD11b 5%
Monocytes (CD11b)
B cells (CD20)
T cells (CD3)
Cell type abundance
CD11b+ [%]
CD20+ [%]
CD3+ [%]
uninjected
no Antibody
αCD5
VSV–G
uninjected
VSV-Gdm+SNAP (no antibody)
VSV-Gdm+SNAP+αCD5-BG
VSV–G
b
Transduction efficiency
total tdTomato+ [%]
tdTomato+ of all CD11b+ [%]
tdTomato+ of all CD20+ [%]
tdTomato+ of all CD3+ [%]
c
FSC-A :: FSC-A x FL5-A :: APC-A750-A
live 75.8
FSC-A :: FSC-A x SSC-A :: SSC-A
Cells 97.6
FSC-A :: FSC-A x FSC-H :: FSC-H
Single Cells 97.8
SSC-A :: SSC-A x SSC-H :: SSC-H
Single Cells 98.9
CD11b :: PB450-A
CD20 :: PB450-A
CD3 :: PB450-A
tdTomato :: PE-A
d
uninjected
VSV-Gdm+SNAP (no antibody)
VSV-Gdm+SNAP+αCD5-BG
VSV–G
αtdTomato
αCD5
αF4/80
DAPI
100μm
e
VSV-Gdm+SNAP+αCD5-BG

Supplementary Figure 8: **Performance of DIRECTED for in vivo T cell targeting**

a, (top) Flowchart depicting the experimental setup for the in vivo evaluation of T cell targeting using αCD5-DIRECTED CreVLPs in Ai14 animals. (bottom) Pie chart showing the cell type composition of murine spleens. (right) Percentage of major cell types (CD11b+, CD19+, CD3+) in splenocytes isolated from mice injected with the indicated vectors. **b,** (left) Overall tdTomato+ cells in splenocytes from animals treated with the indicated vectors. (right) Percentage of tdTomoato+ cells in different subsets of splenocytes. **c,** Gating strategy for the analysis of tdTomato+ cells in different subsets of splenocytes. **d,** Immunofluorescence analysis on spleen slices from animals injected with the indicated vectors stained with antibodies against tdTomato (red), CD5 (white), and F4/80 (green). The slices were counterstained with DAPI to visualize nuclei. (top) Merged visualization of the channels. (bottom) Single channel images. (Representative images of 3 sections) **e,** Merge of the F4/80 (green) and tdTomato (red) staining for the spleen of an animal injected with the VSV-Gdm+SNAP+αCD5-BG vector. Grey arrowheads highlight cells that are positive for F4/80 and tdTomato. (Representative images of 3 sections) For analyses in panels a, and b two-sided Welch's t test was used. [for a, and b, N=2 uninjected animals; N=4 αCD5-SNAP-DIRECTED CreVLPs; N=5 SNAP-DIRECTED CreVLPs; N=6 VSV-G pseudotyped CreVLPs; Scale bar is 100μm] Data are presented as mean ± standard deviation. Source data are provided as a Source Data file.
