# Supplementary material for: Cell type-specific delivery by modular envelope design
Source: Nat Commun. 2023 Aug 23;14:5141. doi: 10.1038/s41467-023-40788-8 (PMC10447438; doi:10.1038/s41467-023-40788-8)
Supplement: Supplementary file 2 — Reporting Summary [file 41467_2023_40788_MOESM2_ESM.pdf]

nature portfolio

Corresponding author(s): Feng Zhang

Last updated by author(s): Jul 28, 2023

Reporting Summary

Nature Portfolio wishes to improve the reproducibility of the work that we publish. This form provides structure for consistency and transparency in reporting. For further information on Nature Portfolio policies, see our Editorial Policies and the Editorial Policy Checklist.

Statistics

For all statistical analyses, confirm that the following items are present in the figure legend, table legend, main text, or Methods section.

n/a	Confirmed	
☐	☒	The exact sample size (*n*) for each experimental group/condition, given as a discrete number and unit of measurement
☐	☒	A statement on whether measurements were taken from distinct samples or whether the same sample was measured repeatedly
☐	☒	The statistical test(s) used AND whether they are one- or two-sided<br>*Only common tests should be described solely by name; describe more complex techniques in the Methods section.*
☒	☐	A description of all covariates tested
☐	☒	A description of any assumptions or corrections, such as tests of normality and adjustment for multiple comparisons
☐	☒	A full description of the statistical parameters including central tendency (e.g. means) or other basic estimates (e.g. regression coefficient) AND variation (e.g. standard deviation) or associated estimates of uncertainty (e.g. confidence intervals)
☐	☒	For null hypothesis testing, the test statistic (e.g. *F*, *t*, *r*) with confidence intervals, effect sizes, degrees of freedom and *P* value noted<br>*Give P values as exact values whenever suitable.*
☒	☐	For Bayesian analysis, information on the choice of priors and Markov chain Monte Carlo settings
☒	☐	For hierarchical and complex designs, identification of the appropriate level for tests and full reporting of outcomes
☒	☐	Estimates of effect sizes (e.g. Cohen's *d*, Pearson's *r*), indicating how they were calculated

Our web collection on statistics for biologists contains articles on many of the points above.

Software and code

Policy information about availability of computer code

Data collection	Flow Cytometry data was acquired using a Cytoflex S instrument running CytExpert (v.2.5); Confocal images were acquired on a Leica DMI8 equipped with a Stellaris 5 camera running Leica Application Suite X (1.4.3)
Data analysis	Flow Cytometry data was analyzed using FlowJo (v 10.8.1). Statistical analyses were performed in R (v 4.2.2 (2022-10-31)) with rstatix package (v 0.7.2), stats package (v 4.3.0), DescTools package (v 0.99.49). Images were analyzed in Fiji (ImageJ2 (v.2.9.0/1.53t). Position-Specific Iterated BLAST (https://blast.ncbi.nlm.nih.gov/Blast.cgi?CMD=Web&PAGE=Proteins&PROGRAM=blastp&RUN_PSIBLAST=on). CD-HIT (http://weizhong-lab.ucsd.edu/cdhit_suite/cgi-bin/index.cgi?cmd=cd-hit, discontinued as of October 2022). Geneious Prime (https://www.geneious.com/prime/, v 2022.2), MAFFT Alignment plugin in Geneious Prime containing L-INS-i Algorithm (v 1.5.0). iTOL (https://itol.embl.de/ v6).

For manuscripts utilizing custom algorithms or software that are central to the research but not yet described in published literature, software must be made available to editors and reviewers. We strongly encourage code deposition in a community repository (e.g. GitHub). See the Nature Portfolio guidelines for submitting code & software for further information.

Data

Policy information about availability of data

All manuscripts must include a data availability statement. This statement should provide the following information, where applicable:

- Accession codes, unique identifiers, or web links for publicly available datasets
- A description of any restrictions on data availability
- For clinical datasets or third party data, please ensure that the statement adheres to our policy

The NCBI non-redundant protein sequences (nr) database can be found at https://blast.ncbi.nlm.nih.gov/Blast.cgi?PROGRAM=blastp&PAGE_TYPE=BlastSearch&LINK_LOC=blasthome. All other data supporting the findings of this study are available within the paper and its Supplementary Information. Source data are provided with this paper.

Research involving human participants, their data, or biological material

Policy information about studies with human participants or human data. See also policy information about sex, gender (identity/presentation), and sexual orientation and race, ethnicity and racism.

Reporting on sex and gender	No human research participants
Reporting on race, ethnicity, or other socially relevant groupings	No human research participants
Population characteristics	No human research participants
Recruitment	No human research participants
Ethics oversight	No human research participants

Note that full information on the approval of the study protocol must also be provided in the manuscript.

Field-specific reporting

Please select the one below that is the best fit for your research. If you are not sure, read the appropriate sections before making your selection.

☒ Life sciences ☐ Behavioural & social sciences ☐ Ecological, evolutionary & environmental sciences

For a reference copy of the document with all sections, see nature.com/documents/nr-reporting-summary-flat.pdf

Life sciences study design

All studies must disclose on these points even when the disclosure is negative.

Sample size	For all in vitro transduction experiments at least three independent infections were performed to ensure reproducibility. No sample-size calculations were performed.<br>For in vivo experiments, we estimated the sample sizes based on relevant publications (e.g., https://doi.org/10.1016/j.cell.2021.08.028, 10.1128/JVI.02032-12).
Data exclusions	no data was excluded.
Replication	All attempts at replication were successful. All in vitro experiments were performed in at least three independent biological replicates.
Randomization	Mice were randomly assigned to the different treatment groups.
Blinding	No blinding was used as all collected data were quantifiable and did not require subjective interpretation.

Reporting for specific materials, systems and methods

We require information from authors about some types of materials, experimental systems and methods used in many studies. Here, indicate whether each material, system or method listed is relevant to your study. If you are not sure if a list item applies to your research, read the appropriate section before selecting a response.

Materials & experimental systems

n/a	Involved in the study
	[x] Antibodies
	[x] Eukaryotic cell lines
[x]	Palaeontology and archaeology
	[x] Animals and other organisms
[x]	Clinical data
[x]	Dual use research of concern
[x]	Plants

Methods

n/a	Involved in the study
[x]	ChIP-seq
	[x] Flow cytometry
[x]	MRI-based neuroimaging

Antibodies

Antibodies used

αHLA-A2, BioLegend, 343302, Clone BB7.2;
αCD3, BioLegend, 317302, Clone OKT3;
αCD5, BioLegend, 300602, Clone UCHT2;
αCD46, BioLegend, 352403, Clone TRA-2-10;
αVSV-G C-term, Millipore Sigma, V5507-.2ML, Clone P5D4;
αCre, Cell Signaling Technology, 15036S, Clone D7L7L;
αHA, BioLegend, 901502, Clone 16B12;
αSpot, ChromoTek, 28a5-100, Clone 28A5;
αV5, Thermo Fisher Scientific, X2720M, Clone E10;
αStrep, Qiagen, 34850;
αB2M, BioLegend, 316302, Clone 2M2;
αCD117, Stem Cell Technologies, 60087, Clone 104D2;
αmouse-FITC, BioLegend, 406001, Clone Poly4060;
αmouse-680RD, LiCor, 926-68020, Polyclonal;
αrat-800CW, LiCor, 926-32219, Polyclonal;
αrabbit-800CW, Licor, 926-32211, Polyclonal;
αHLA-ClassI, Abcam, ab23755, Clone W6/32;
αB2M-FITC, BioLegend, 316304, Clone 2M2;
αCD4, BioLegend, 317402, OKT4;
αCD19, BioLegend, 302202, HIB19;
αCD28, BioLegend, 302933, CD28.2;
Human Fc block, BioLegend, 422302;
Mouse Fc block, BD Biosciences, 553142;
αCD3-BV421 (mouse), BioLegend, 100228, 17A2;
αCD20-BV421 (mouse), BioLegend, 150405, SA275A11;
αCD11b-BV421 (mouse), BioLegend, 101236, M1/70;
αRFP-568, NanoTag Biotechnologies, N0404;
αF4/80-AF647, BioLegend, 123122, BM8;
αCD5-FITC, BioLegend, 100606, 53-7.3;
αCD4-FITC, BioLegend, 300506, RPA-T4;
αCD3-PerCP/Cy5.5, BioLegend, 300328, HIT3a;
αCD8-PB, BioLegend, 301033, RPA-T8;
αCD19-APC, BioLegend, 302212, HIB19;
αCD14-AF647, BioLegend, 301818, M5E2;
αCD19-BV510, BioLegend, 302242, HIB19.

Validation

αHLA-A2, BioLegend, 343302, Clone BB7.2: Each lot of this antibody is quality control tested by immunofluorescent staining with flow cytometric analysis by BioLegend. Additionally, we tested the antibody on HEK293FT cells and confirmed specificity in B2M knockout HEK293FT cells.
αCD3, BioLegend, 317302, Clone OKT3: Each lot of this antibody is quality control tested by immunofluorescent staining with flow cytometric analysis by BioLegend. Additionally, we tested this antibody to stain Jurkat E6 cells, which are known to express CD3.
αCD5, BioLegend, 300602, Clone UCHT2: Each lot of this antibody is quality control tested by immunofluorescent staining with flow cytometric analysis by BioLegend. Additionally, we tested this antibody to stain Jurkat E6 cells, which are known to express CD3.
αCD46, BioLegend, 352403, Clone TRA-2-10: Each lot of this antibody is quality control tested by immunofluorescent staining with flow cytometric analysis by BioLegend.
αVSV-G C-term, Millipore Sigma, V5507-.2ML, Clone P5D4: This antibody was tested for performance in Western Blots on E.Coli or 293T Cells Exp VSV-G Tag lysates by Millipore Sigma.
αCre, Cell Signaling Technology, 15036S, Clone D7L7L: used in multiple publications (e.g., https://pubmed.ncbi.nlm.nih.gov/9288963/)
αHA, BioLegend, 901502, Clone 16B12: Each lot of this antibody is quality control tested by Western Blotting by BioLegend. Moreover, in our experiments where we use multiple cell lines expressing synthetic receptors, this antibody only stains cells that express the Surface-HA construct as evaluated by Flow Cytometry.
αSpot, ChromoTek, 28a5-100, Clone 28A5: The specificity of the antibody is evaluated by Western Blotting using Spot-tagged GFP (N-

or C-terminal) added to HEK-293T cell lysate by the supplier. Moreover, in our experiments where we use multiple cell lines expressing synthetic receptors, this antibody only stains cells that express the Surface-Spot construct as evaluated by Flow Cytometry.
αV5, Thermo Fisher Scientific, X2720M, Clone E10: It is validated for Dot Blot, ELISA, Immunoprecipitation, Immunoassay, Western Blot by the Supplier. Moreover, in our experiments where we use multiple cell lines expressing synthetic receptors, this antibody only stains cells that express the Surface-V5 construct as evaluated by Flow Cytometry.
αStrep, Qiagen, 34850: Used in multiple publications (e.g., https://pubmed.ncbi.nlm.nih.gov/32353859/). Moreover, in our experiments where we use multiple cell lines expressing synthetic receptors, this antibody only stains cells that express the Surface-Strep construct as evaluated by Flow Cytometry.
αB2M, BioLegend, 316302, Clone 2M2: Each lot of this antibody is quality control tested by immunofluorescent staining with flow cytometric analysis by BioLegend.
αCD117, Stem Cell Technologies, 60087, Clone 104D2: This antibody clone has been verified for purity assessments of cells cultured with STEMdiff™ Definitive Endoderm Kit by the supplier.
αmouse-FITC, BioLegend, 406001, Clone Poly4060: Each lot of this antibody is quality control tested by immunofluorescent staining with flow cytometric analysis by BioLegend.
αmouse-680RD, LiCor, 926-68020, Polyclonal: This antibody was tested by Dot Blot and/or solid-phase adsorbed for minimal crossreactivity with human, rabbit, goat, rat, and horse serum proteins, but may cross-react with immunoglobulins from other species. The conjugate has been specifically tested and qualified for Western blot.
αrat-800CW, LiCor, 926-32219, Polyclonal: This antibody was tested by Dot Blot and/or solid-phase adsorbed for minimal crossreactivity with human, rabbit, goat, rat, and horse serum proteins, but may cross-react with immunoglobulins from other species. The conjugate has been specifically tested and qualified for Western blot.
αrabbit-800CW, Licor, 926-32211, Polyclonal: This antibody was tested by Dot Blot and/or solid-phase adsorbed for minimal crossreactivity with human, rabbit, goat, rat, and horse serum proteins, but may cross-react with immunoglobulins from other species. The conjugate has been specifically tested and qualified for Western blot.
αHLA-ClassI, Abcam, ab23755, Clone W6/32: used in previous publications (e.g., https://pubmed.ncbi.nlm.nih.gov/32276457/).
αB2M-FITC, BioLegend, 316304, Clone 2M2: Each lot of this antibody is quality control tested by immunofluorescent staining with flow cytometric analysis by BioLegend.
αCD4, BioLegend, 317402, OKT4: Each lot of this antibody is quality control tested by immunofluorescent staining with flow cytometric analysis by BioLegend.
αCD19, BioLegend, 302202, HIB19: Each lot of this antibody is quality control tested by immunofluorescent staining with flow cytometric analysis by BioLegend.
αCD28, BioLegend, 302933, CD28.2: Each lot of this antibody is quality control tested by immunofluorescent staining with flow cytometric analysis by BioLegend.
αCD3-BV421 (mouse), BioLegend, 100228, 17A2: Each lot of this antibody is quality control tested by immunofluorescent staining with flow cytometric analysis by BioLegend.
αCD20-BV421 (mouse), BioLegend, 150405, SA275A11: Each lot of this antibody is quality control tested by immunofluorescent staining with flow cytometric analysis by BioLegend.
αCD11b-BV421 (mouse), BioLegend, 101236, M1/70: Each lot of this antibody is quality control tested by immunofluorescent staining with flow cytometric analysis by BioLegend.
αRFP-568, NanoTag Biotechnologies, N0404: no additional validation
αF4/80-AF647, BioLegend, 123122, BM8: Each lot of this antibody is quality control tested by immunofluorescent staining with flow cytometric analysis by BioLegend.
αCD5-FITC, BioLegend, 100606, 53-7.3: Each lot of this antibody is quality control tested by immunofluorescent staining with flow cytometric analysis by BioLegend.
αCD4-FITC, BioLegend, 300506, RPA-T4: Each lot of this antibody is quality control tested by immunofluorescent staining with flow cytometric analysis by BioLegend.
αCD3-PerCP/Cy5.5, BioLegend, 300328, HIT3a: Each lot of this antibody is quality control tested by immunofluorescent staining with flow cytometric analysis by BioLegend.
αCD8-PB, BioLegend, 301033, RPA-T8: Each lot of this antibody is quality control tested by immunofluorescent staining with flow cytometric analysis by BioLegend.
αCD19-APC, BioLegend, 302212, HIB19: Each lot of this antibody is quality control tested by immunofluorescent staining with flow cytometric analysis by BioLegend.
αCD14-AF647, BioLegend, 301818, M5E2: Each lot of this antibody is quality control tested by immunofluorescent staining with flow cytometric analysis by BioLegend.
αCD19-BV510, BioLegend, 302242, HIB19: Each lot of this antibody is quality control tested by immunofluorescent staining with flow cytometric analysis by BioLegend.

Eukaryotic cell lines

Policy information about cell lines and Sex and Gender in Research

Cell line source(s)	HEK293FT: Thermo Fisher Scientific (Cat. # R70007)<br>Jurkat E6: ATCC (Cat. # TIB152)<br>A549+Ace2: gift from Prof. Gewurz, initially from ATCC (Cat. # CCL-185)<br>Kasumi-1: ATCC (Cat. # CRL-2724)<br>OUMS-23: Genetic Perturbation Platform (GPP) at the Broad Institute<br>HepG2: ATCC (Cat. # HB-8065)<br>K562+HLA-A2+eGFP: gift from Prof. Baltimore, initially from ATCC (Cat. # CRL-3343)
Authentication	None of the cell lines were additionally authenticated

Mycoplasma contamination	All cell lines tested negative for mycoplasma contamination
Commonly misidentified lines (See ICLAC register)	None of the cell lines used are listed in the ICLAC register

Animals and other research organisms

Policy information about studies involving animals; ARRIVE guidelines recommended for reporting animal research, and Sex and Gender in Research

Laboratory animals	4–6-week-old female Ai14 mice (B6.Cg-Gt(ROSA)26Sortm14(CAG-tdTomato)Hze/J; JAX Strain #:007914, N=17)
Wild animals	Study did not involve wild animals.
Reporting on sex	No sex based analysis has been performed.
Field-collected samples	Study did not involve samples collected from the field.
Ethics oversight	All experiments were approved by the Institutional Animal Care and Use Committee of the Broad Institute (Protocol ID 0017-09-14-2). Animal maintenance and handling complied with all relevant ethical regulations.

Note that full information on the approval of the study protocol must also be provided in the manuscript.

Flow Cytometry

Plots

Confirm that:

- [x] The axis labels state the marker and fluorochrome used (e.g. CD4-FITC).
- [x] The axis scales are clearly visible. Include numbers along axes only for bottom left plot of group (a 'group' is an analysis of identical markers).
- [x] All plots are contour plots with outliers or pseudocolor plots.
- [x] A numerical value for number of cells or percentage (with statistics) is provided.

Methodology

Sample preparation	All flow cytometry experiments with fluorescent proteins were performed as follows: For adherent cells, media was initially removed, and cells were washed once with PBS (Thermo Fisher Scientific, Cat. # 10010049), before they were released from the dish using TrypLE (Thermo Fisher Scientific, Cat. # 12604021). After 5 minutes the reaction was stopped by resuspending the cells in DMEM with 10% FBS and Pen/Strep and the cell suspension was transferred to a V-bottom 96-well plate before harvesting the cells by centrifugation (1000xg, 3 minutes). The cells were washed twice with Flow Buffer (PBS+2% FBS+5 mM EDTA) containing DAPI (100 ng/ml; Thermo Fisher Scientific, Cat. # D1306) before resuspending in Flow Buffer (without DAPI) and analyzed on a Beckman Coulter CytoFLEX S device. Suspension cells were triturated to break up cell clumps, before immediately being transferred to a V-bottom plate and harvested by centrifugation (1000xg, 3 minutes). As above, cells were washed twice with Flow Buffer containing 100 ng/ml DAPI, and finally resuspended in Flow Buffer before being analyzed on a CytoFLEX S device. All data was acquired using CytExpert software and analyzed using FlowJo (https://www.flowjo.com/).<br>For staining of surface receptors, cells were treated as described above, with an additional incubation of cells with an antibody after the first wash in Flow Buffer. The used antibodies and corresponding dilutions are listed in table of antibodies. In case of fluorophore spillover, unstained and single stained samples were acquired and used to calculate the necessary compensation in FlowJo.
Instrument	Beckman Coulter, Cytoflex S
Software	Data acquisition: CytExpert (v.2.5)<br>Data analysis: FlowJo (v 10.8.1)
Cell population abundance	HEK293FT ΔB2M were stained with αB2M-FITC, sorted for low B2M and purity verified by flow cytometry to be higher than 95%. For Jurkat E6 + surface-HA, cells were stained with αHA-PB450 and sorted into four bins of different expression levels. The purity of these bins was confirmed by postsort runs, confirming at least 50% of cells falling into the specified bins. The effect of peak broadening was more pronounced for the surface-HA medium low and medium high bins. These cells were additionally evaluated by flow cytometry before their use in experiments and confirmed different mean expression levels, despite some overlap in expression levels (compare Supplementary Figure 2f.

Gating strategy	Gating strategies are highlighted in the supplementary figures. For most flow cytometry analysis cells were initially gated by FSC-A vs SSC-A to exclude debris, before gating in SSC-A vs SSC-H for Single Cells. The single cell population was then gated for live cells using PB450-A vs FSC-A to exclude cells that were staining positive with DAPI. Finally, this live cell gate was plotted for the markers of interest (e.g., antibody staining or lentivirally delivered H2B-mCherry+).<br>For the K562 and Jurkat mixing experiment the live cell population was plotted in a FITC-A vs FSC-A plot where GFP+ (FITC-A high cells) were identified as K562 cells. Subsequently, the fraction of H2B-mCherry+ cells was analyzed in the GFP- cells (Jurkat E6) and in the GFP+ cells (K562).

☒ Tick this box to confirm that a figure exemplifying the gating strategy is provided in the Supplementary Information.
