# Supplementary material for: Cell type-specific delivery by modular envelope design
Source: Nat Commun. 2023 Aug 23;14:5141. doi: 10.1038/s41467-023-40788-8 (PMC10447438; doi:10.1038/s41467-023-40788-8)
Supplement: Supplementary file 3 — Description of Additional Supplementary Files [file 41467_2023_40788_MOESM3_ESM.pdf]

Title: Supplementary Data 1: Overview of Fusogen library
Description: Table showing the IDs, used throughout the main text, and the corresponding Name and Family from which these proteins were derived.

Title: Supplementary Data 2: Overview of plasmids and oligonucleotides
Description: Excel file containing information on the plasmids generated in this study and the oligonucleotides used.

Title: Supplementary Data 3: Overview of antibodies
Description: Antibodies used in this study and corresponding dilutions.
